# MOVECLIM–AZORES: plot based vascular plant cover along elevational gradients (2013)

**DOI:** 10.3897/BDJ.14.e178119

**Published:** 2026-03-25

**Authors:** Rui Andrade, Rosalina Gabriel, Paulo A. V. Borges, Rui Bento Elias

**Affiliations:** 1 University of Azores, CE3C—Centre for Ecology, Evolution and Environmental Changes, Azorean Biodiversity Group, CHANGE —Global Change and Sustainability Institute, School of Agricultural and Environmental Sciences, Rua Capitão João d’Ávila, Pico da Urze, 9700-042, Angra do Heroísmo, Azores, Portugal University of Azores, CE3C—Centre for Ecology, Evolution and Environmental Changes, Azorean Biodiversity Group, CHANGE —Global Change and Sustainability Institute, School of Agricultural and Environmental Sciences, Rua Capitão João d’Ávila, Pico da Urze, 9700-042 Angra do Heroísmo, Azores Portugal; 2 IUCN SSC Monitoring Specialist Group, Angra do Heroísmo, Azores, Portugal IUCN SSC Monitoring Specialist Group Angra do Heroísmo, Azores Portugal

**Keywords:** Azores, biodiversity monitoring, islands, vascular plants, endemism, invasive species, occurrences, field survey, long-term monitoring, Wallacean shortfall

## Abstract

**Background:**

In the scope of the MOVECLIM project, in 2013, we surveyed vascular plants and their ground cover in native or better-preserved vegetation patches, following an elevational transect, on four Azorean islands (São Miguel, Terceira, Pico and Flores). Using a standardised, plot-based protocol, permanent 10 m × 10 m plots were established at approximately 200 m elevation steps, each plot subdivided into four 5 m × 5 m subplots. All vascular taxa were recorded per subplot and cover was assigned using the Braun-Blanquet Ordinal Transform Value (OTV), a scale based on a geometric series, that assigns a numeric value (1–9) to each Braun–Blanquet category.

**New information:**

We established 58 permanent plots across the four Azorean islands, resulting in 232 event records at the subplot level (eventID) and 2,539 occurrence records (occurrenceID), spanning 58 families, 92 genera, 109 species and five subspecies of vascular plants. Amongst these taxa, 44 are Azorean endemics, 40 are native non-endemic and 30 are introduced (of which 17 are invasive). This study provides a baseline for long-term monitoring of native vegetation, assessment of invasive species and evaluation of native communities' responses to such pressures across islands with contrasting elevation ranges and disturbance histories.

## Introduction

Long-term ecological monitoring is essential to detect shifts in community composition, set conservation priorities and understand global-change impacts (Scheele et al. 2019, Lindenmayer et al. 2022). Elevational gradients are particularly informative in this regard, because they capture environmental variation over short spatial distances and can serve as early indicators of climate-driven shifts (Colwell and Lees 2000, Kluge et al. 2006).

Although several floristic and ecological studies have been conducted in the Azores, particularly in protected areas, systematic elevational surveys of vascular plant diversity remain relatively scarce. Here, we aim to fill this gap by presenting a multi-island, plot-based dataset, designed to support the long-term study of vascular plant diversity across the Azores.

## General description

### Purpose

To publish a standardised baseline of vascular plant occurrences and percentage cover along elevational gradients on four Azorean islands, enabling long-term monitoring and cross-island comparisons.

## Project description

### Title

MOVECLIM–AZORES: plot-based vascular plant cover across elevational gradients in Azorean native forests (2013)

### Personnel

The vascular plant inventory and identification was carried out by Rui Bento Elias (RBE), with the participation of Fernando Pereira (FP). Databases were compiled by Rui Andrade (RA). Darwin-Core data were verified and managed in GBIF IPT by Paulo A.V. Borges.

### Study area description

**Figure 1.**
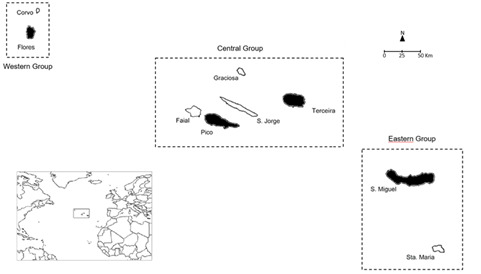
The Azores Archipelago is located in the middle North Atlantic (left panel). The Azorean Islands covered in this study, São Miguel, Terceira, Pico and Flores are highlighted in black.

The Azores Archipelago (Portugal) comprises nine volcanic islands in the North Atlantic Ocean (36º55’–39º43’ N; 24º46’–31º16’ W), ranging in age from 0.3 to 8.1 million years (França et al. 2003) (see Fig. 1) and grouped into Western (Corvo, Flores), Central (Faial, Pico, Graciosa, São Jorge, Terceira) and Eastern (São Miguel, Santa Maria) groups. The Archipelago lies approximately 1,600 km west of mainland Portugal and has a total land area of 2,332.7 km² (Forjaz 2004).

The climate is temperate oceanic, with high humidity, regular and abundant rainfall, mild temperatures and frequent strong winds (Azevedo et al. 2004).

### Design description

This research comprised four islands of the three main groups of the Azores Archipelago: São Miguel Island with 745 km^2^, the highest elevation being 1105 m a.s.l. with an estimated age of 0.79 MY (millions of years) (Sibrant et al. 2015); Terceira Island with 400 km^2^, a maximum elevation of 1023 m a.s.l. and 0.39 MY (Hildenbrand et al. 2014); Pico Island with an area of 447 km^2^, mostly occupied by volcanoes reaching an altitude of 2351 m a.s.l. and an approximate age of 0.27 MY (Demand et al. 1982); and Flores Island with an area of 143 km^2^, the highest elevation being 914 m a.s.l., with an estimated age of 2.2 MY (Azevedo and Ferreira 2006).

Following Elias et al. (2016), Azorean native vegetation is arranged in altitudinal belts. Our 200-m elevation transects deliberately cut across these belts, ensuring that plots capture the main vegetation types per island.

Centuries of land-use change and biological invasions have extensively transformed Azorean native vegetation (Dias 2007). Invasives exert marked pressure, particularly on São Miguel, where native remnants are often heavily encroached by species, such as *Pittosporum undulatum*, *Clethra arborea* and *Hedychium gardnerianum* (Moniz and Silva 2003, Hortal et al. 2010). The remaining native forest mosaics are thus most intact in the higher, wetter belts, characterised by cloud-forest conditions.

### Funding

This study was originally financed by ERANET BIOME MOVECLIM ‘Montane vegetation as listening posts for climate change’ of the Regional Government of the Azores, grant number M2.1.2/F/04/2011/NET. RA received a Ph.D. Grant from FCT (UIDB/00329/2020-2024; DOI: 10.54499/UIDB/00329/2020), under Thematic Line 1 – Integrated ecological assessment of environmental change on biodiversity. While working on this publication, RBE, RG and PAVB were funded by FCT through national and European funds by UID/00329/2025 - Centre for Ecology, Evolution and Environmental Change (CE3C). This study is part of the Biodiversa+ project BioMonI – Biodiversity monitoring of island ecosystems, funded by FCT (BiodivMon/0003/2022), which also supported Open Access and provided funding to RG, RBE and PAVB.

## Sampling methods

### Sampling description

**Figure 2.**
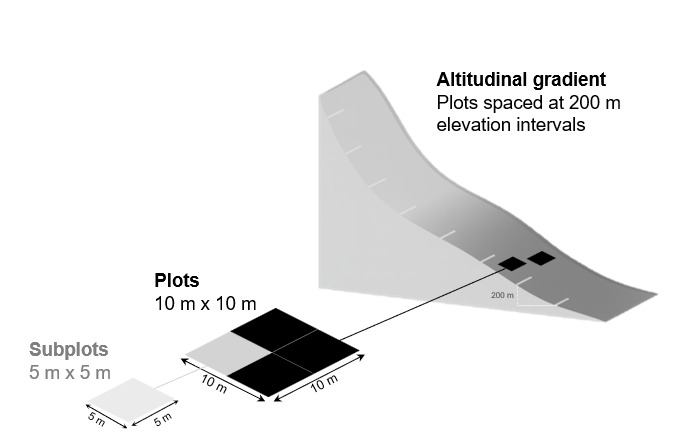
Altitudinal sampling model on all islands (adapted from Ah-Peng et al. (2012), Gabriel et al. (2014)): at 200 m elevation steps, two plots (black squares, 10 m × 10 m) were placed within 10 m to 15 m from each other; each plot is subdivided into four subplots of 5 m × 5 m (grey squares); all vascular plant species were recorded and their cover assigned to ordinal OTV classes (1-9).

On each of the four islands, at each elevation level within areas dominated by native vegetation, two permanent 10 m × 10 m plots were established at approximately 200 m intervals, ranging from sea level to the highest summit. The two plots at each elevation were placed 10–15 m apart to minimise microhabitat overlap. Each plot was subdivided into four 5 m × 5 m subplots. All vascular plant species present were recorded within each subplot following an adapted BRYOLAT protocol (Ah-Peng et al. 2012, Gabriel et al. 2014), designed for standardised sampling across altitudinal gradients (Fig. 2) and presently known as the GIMS protocol (Borges et al. 2018).

For each species, cover was estimated using the OTV scale (van der Maarel 1979): 1 (≤ 0.5%), 2 (0.5–1.5%), 3 (1.5–3%), 4 (3–5%), 5 (5–12.5%), 6 (12.5–25%), 7 (25–50%), 8 (50–75%), 9 (≥ 75%).

The sampling locations and coordinates are listed in Table 1.

### Quality control

In the field, identifications were checked with an Azorean flora guide (Schaefer 2005).

Taxonomic nomenclature follows the Azorean Biodiversity Portal vascular flora backbone (ABP 2025) together with regional checklists (Borges et al. 2010, Silva et al. 2010, Gabriel and Borges 2022, Silva et al. 2022).

The colonisation status of each species was determined, based on its biogeographical origin and degree of naturalisation in the Azores (Silva et al. 2008, Martín et al. 2008, ABP 2025).

## Geographic coverage

### Description

This study was carried out in São Miguel (Eastern group), Terceira and Pico (Central group) and Flores (Western group) islands of the Azorean Archipelago.

### Coordinates

37.551908 and 39.650021 Latitude; -31.378349 and -24.822498 Longitude.

## Taxonomic coverage

### Description

**Phylum**: Tracheophyta

**Classes**: Selaginellopsida, Polypodiopsida, Pinopsida, Liliopsida, Magnoliopsida

## Temporal coverage

### Notes

Fieldwork was performed from 1 March to 29 September 2013.

## Usage licence

### Usage licence

Creative Commons Public Domain Waiver (CC-Zero)

## Data resources

### Data package title

The MOVECLIM – AZORES project: Vascular Plant Diversity and Ground Cover Across Elevation (São Miguel, Terceira, Pico & Flores)

### Resource link

https://doi.org/10.15468/qs5rpt

### Alternative identifiers

https://ipt.gbif.pt/ipt/resource?r=plants_plots_moveclim; https://www.gbif.org/dataset/f92047fa-f2cb-445d-9a93-546d637b2820

### Number of data sets

2

### Data set 1.

#### Data set name

Event Table

#### Data format

Darwin Core Archive format

#### Character set

UTF-8

#### Download URL

https://ipt.gbif.pt/ipt/resource?r=plants_plots_moveclim

#### Data format version

Version 1.6

#### Description

The dataset was published on the Global Biodiversity Information Facility platform, GBIF (Andrade et al. 2025). The dataset submitted to GBIF is structured as a sample event dataset published as a Darwin Core Archive (DwCA), a standardised format for sharing biodiversity data as a set of one or more data tables. The core data file contains 232 records at the subplot level (eventID). This GBIF IPT (Integrated Publishing Toolkit, Version 2.5.6) archives the data and, thus, serves as the data repository. The data and resource metadata are available for download in the Portuguese GBIF Portal IPT (Andrade et al. 2025).

**Data set 1.** 

Column label	Column description
datasetName	The name identifying the dataset from which the record was derived.
eventID	Identifier of the events, unique for the dataset.
samplingProtocol	The sampling protocol used to record the species.
sampleSizeValue	The numeric amount of the sampling area.
sampleSizeUnit	The unit of the sample size value.
eventDate	Date of the sampling.
startDayOfYear	The earliest integer day of the year on which the event occurred.
day	The day in which the event occurred.
month	The month in which the event occurred.
year	The year in which the event occurred.
habitat	The habitat for an Event.
locationID	Identifier of the location.
islandGroup	Name of the archipelago.
island	Name of the island.
country	Country of the sampling site.
countryCode	The standard code for the country of the sampling site.
stateProvince	Name of the region of the sampling site.
municipality	Municipality of the sampling site.
locality	Name of the locality.
minimumElevationInMetres	The lower limit of the range of elevation (altitude, usually above sea level), in metres.
maximumElevationInMetres	The upper limit of the range of elevation (altitude, usually above sea level), in metres.
locationRemarks	Comments or notes about the Location.
decimalLatitude	Approximate centre point decimal latitude of the field site in GPS coordinates.
decimalLongitude	Approximate centre point decimal longitude of the field site in GPS coordinates.
geodeticDatum	The geodetic datum or spatial reference system (SRS), upon which the geographic coordinates given in decimalLatitude and decimalLongitude are based.
coordinateUncertaintyInMetres	The horizontal distance (in metres) from the given decimal Latitude and decimal Longitude describing the smallest circle containing the whole of the location.
verbatimCoordinates	Original coordinates recorded.
coordinatePrecision	Value in decimal degrees to a precision of five decimal places.
georeferenceSources	Method used to obtain coordinates.

### Data set 2.

#### Data set name

Occurrence Table

#### Data format

Darwin Core

#### Character set

UTF-8

#### Download URL

https://ipt.gbif.pt/ipt/resource?r=plants_plots_moveclim

#### Data format version

Version 1.6

#### Description

The dataset was published on the Global Biodiversity Information Facility platform, GBIF (Andrade et al. 2025). The dataset submitted to GBIF is structured as an occurrence table and published as a Darwin Core Archive (DwCA), which is a standardised format for sharing biodiversity data as a set of one or more data tables. The core data file contains 2,538 records (occurrenceID). This GBIF IPT (Integrated Publishing Toolkit, Version 2.5.6) archives the data and, thus, serves as the data repository. The data and resource metadata are available for download in the Portuguese GBIF Portal IPT (Andrade et al. 2025). All taxa were identified to species/subspecies level, with the exception of Potentilla sp.

**Data set 2.** 

Column label	Column description
licence	Reference to the licence under which the record is published.
institutionID	The identity of the institution publishing the data.
institutionCode	The code of the institution publishing the data.
basisOfRecord	The nature of the data record.
occurrenceID	Identifier of the record, coded as a global unique identifier.
recordedBy	A list of names of the people who performed the sampling in the field.
organismQuantity	Estimated species percent cover.
organismQuantityType	The type of quantification system used for the measure of cover.
establishmentMeans	The process of establishment of the species in the location, using a controlled vocabulary: 'endemic', 'native', 'introduced'.
eventID	Identifier of the events, unique for the dataset.
identifiedBy	A list of names of people who assigned the Taxon to the subject.
dateIdentified	Date on which the record was identified.
scientificName	Complete scientific name including author.
kingdom	Kingdom name.
phylum	Phylum name.
class	Class name.
order	Order name.
family	Family name.
genus	Genus name.
specificEpithet	Specific epithet.
infraspecificEpithet	Infraspecific epithet, when available.
taxonRank	Lowest taxonomic rank of the record.
scientificNameAuthorship	Name of the author of the lowest taxon rank included in the record.

## Additional information

The 232 events across the four islands yielded 2,539 records, encompassing 114 vascular plant taxa, represented by 109 species and five subspecies. The majority of these taxa are indigenous (almost 39% endemic and 35% native), while 26% are non-indigenous (Table 2, Andrade et al. (2025)). Five categories were adopted, listed in order of increasing generality: Azorean endemics (END) — referring to species naturally occurring only in the Azores; native species (NAT) — which colonised the Azores via long-distance dispersal and are also present in other archipelagos or continents; introduced and naturalised species (INT NAT) — introduced through human action and capable of maintaining self-sustaining populations; invasive species (INV) — exotic species that establish themselves in natural or semi-natural ecosystems and cause measurable impacts or threats to native biodiversity.

**Figure 3.**
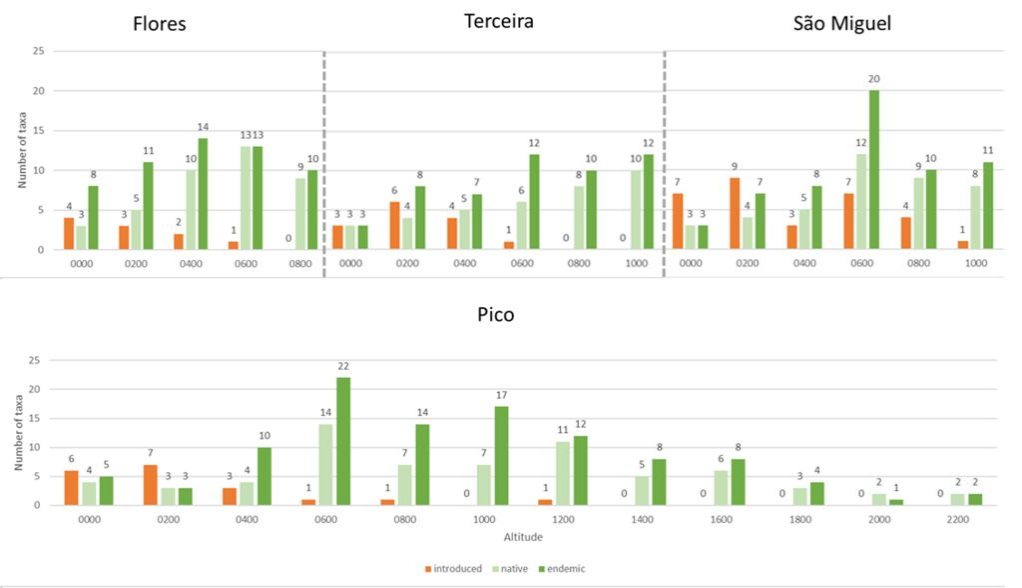
Number of taxa (species and subspecies) along the elevational gradient on Flores (maximum altitude 914 m a.s.l.), Terceira (max. alt. 1,021 m a.s.l.), Pico (max. alt. 2,351 m a.s.l.) and São Miguel (max. alt. 1,103 m a.s.l.) islands, recorded using the MOVECLIM Protocol in 2013, by colonisation status.

Across the four islands, the richness of indigenous species increases from low elevations to a peak around 600 m, followed by a decline towards higher elevations. Introduced species are concentrated at low elevations (0–400 m) and become rare or absent above 600–800 m, a pattern consistent across all islands (Fig. 3).

The recorded species were distributed in five classes, 58 families and 92 genera. The most species-rich families were: Poaceae (10 taxa); Dryopteridaceae, Cyperaceae and Rosaceae (seven taxa each); and Asteraceae (six taxa). Suppl. material 1 provides a per-family list of genera and infrageneric taxa for all recorded vascular plants on the islands covered in this study.

### Conservation and Management

According to the IUCN Red List (IUCN 2025), six species are considered to be of conservation concern: one is Critically Endangered (CR), three are Endangered (EN) and two are Vulnerable (VU). Amongst the remaining indigenous vascular plant taxa, 29 are considered as Least Concern (LC) and two are considered Near Threatened (NT). When comparing our checklist of indigenous species to the regional legislation for species conservation (DLR15 2012), 29.5% of the species are covered by protection and conservation measures and 21% are considered prioritary for conservation (Suppl. material 2).

Regarding the 17 invasive alien species reported in the dataset (cf. Suppl. material 3, ABP (2025)), 10 are included in DLR15 (2012), five are considered priority for control or eradication, while six are thought to pose ecological risks. Furthermore, if we compare our list with the Top 100 invasive species in the Azores (Silva et al. 2008), five of the 17 are in the top 25 (Q1), five in the second quartile (Q2) and one in the third quartile (Q3). Of these 11 species, only *Acacia melanoxylon* R.Br. (Q2), *Adiantum hispidulum* Sw. (Q2) and *Cyrtomium falcatum* (L.f.) C.Presl (Q1) are not included in the DLR15 (2012).

## Supplementary Material

2E10444A-5FD8-56EC-ADD4-49FFB550B143 10.3897/BDJ.14.e178119.suppl1 Supplementary material 1 List of genera and infrageneric taxa recorded, by families. Data type List of taxa occurrences Brief description Number of genera and of infrageneric *taxa* per family, of all vascular plants recorded, on four Azorean Islands (São Miguel, Terceira, Pico and Flores). Numbers within brackets represent the number of genera shared between indigenous (IND - endemic and native) and introduced (INT - introduced naturalised, casual or invasive) taxa. File: oo_1459842.csv https://binary.pensoft.net/file/1459842 Andrade, R

BA0991EF-F782-5A21-9C23-F6A429805CA1 10.3897/BDJ.14.e178119.suppl2 Supplementary material 2 Conservation and management species list Data type Species checklist Brief description Colonisation status according to PBA (2025): END - Azorean endemic; NAT - native. Conservation status according to IUCN (2025): CR - Critically Endangered; EN - Endangered; VU - Vulnerable; NT - Near Threatened; LC - Least Concern. Taxa included on (“X” indicates presence on the indicated list): DLR15/2012/A (regional legislation for species conservation); H (Habitats Directive - Natura 2000 Network); B (Berne Convention); T100 (list of 100 priority threatened species for management in the European biogeographical region of Macaronesia); CITES (Convention on International Trade in Endangered Species of Wild Fauna and Flora); R4 (protected by regional interest); P (priority for conservation). File: oo_1459843.csv https://binary.pensoft.net/file/1459843 Andrade, R.

24F11181-87BC-52F8-A5C5-2CA3C0B40239 10.3897/BDJ.14.e178119.suppl3 Supplementary material 3 Invasive species list Data type Species checklist Brief description Colonisation status according to PBA (2025): INT NAT - introduced naturalised; INV - invasive. Taxa included on (“X” indicates presence on the indicated list): DLR15/2012/A (regional legislation for species conservation), P (priority for control or eradication), R (with ecological risks). The Top 100 (T100) invasive species in Macaronesia (Silva et al. 2008) are represented in quartiles (Q1 (Top 25); Q2 (26-50); Q3 (51-75); Q4 (76-100)). File: oo_1511619.csv https://binary.pensoft.net/file/1511619 Andrade, Rui

## Figures and Tables

**Table 1.** List of the 58 plots established in Flores (n = 10), Pico (n = 24), São Miguel (n = 12) and Terceira (n = 12) islands. Elevation in metres above sea level and coordinates in decimals (datum WGS84).

Island	Plot_code	Municipality	Locality	Elevation (m a.s.l.)	Latitude	Longitude
Flores	FLO_0060_P1	Santa Cruz	Ponta do Ilhéu	75	39.506464	-31.194688
	FLO_0060_P2			55	39.506187	-31.194626
	FLO_0200_P1		Ponta Delgada (200 m)	248	39.506932	-31.212854
	FLO_0200_P2			242	39.506609	-31.212727
	FLO_0400_P1		Ponta Delgada (400 m)	379	39.501848	-31.205639
	FLO_0400_P2			378	39.501847	-31.205623
	FLO_0600_P1		Cedros	647	39.482888	-31.190428
	FLO_0600_P2			643	39.482683	-31.190348
	FLO_0800_P1		Morro Alto	828	39.463191	-31.225955
	FLO_0800_P2			829	39.463200	-31.225988
Pico	PIC_0010_P1	Lajes	Manhenha	16	38.413700	-28.02994
	PIC_0010_P2			16	38.413855	-28.029837
	PIC_0200_P1		Cabeço da Hera	222	38.418127	-28.053985
	PIC_0200_P2			218	38.418193	-28.053614
	PIC_0400_P1		Fetais	356	38.425861	-28.087491
	PIC_0400_P2			362	38.425918	-28.087264
	PIC_0600_P1	São Roque	Chão Verde	624	38.479754	-28.272413
	PIC_0600_P2			629	38.479491	-28.272322
	PIC_0800_P1	Lajes	Caiado	808	38.455640	-28.257293
	PIC_0800_P2			805	38.455504	-28.257308
	PIC_1000_P1		Caveiro	955	38.437177	-28.213032
	PIC_1000_P2			954	38.437170	-28.212779
	PIC_1200_P1	Madalena	Pico Mountain trail (1200 m)	1268	38.470360	-28.425126
	PIC_1200_P2			1265	38.470683	-28.424985
	PIC_1400_P1		Pico Mountain trail (1400 m)	1406	38.469385	-28.421358
	PIC_1400_P2			1406	38.469292	-28.421351
	PIC_1600_P1		Pico Mountain trail (1600 m)	1601	38.465878	-28.416554
	PIC_1600_P2			1596	38.465353	-28.416437
	PIC_1800_P1		Pico Mountain trail (1800 m)	1799	38.465962	-28.412560
	PIC_1800_P2			1800	38.465901	-28.412543
	PIC_2000_P1		Pico Mountain trail (2000 m)	2010	38.465647	-28.408192
	PIC_2000_P2			2012	38.465489	-28.408084
	PIC_2200_P1		Pico Mountain trail (2200 m)	2245	38.466337	-28.399242
	PIC_2200_P2			2248	38.466756	-28.399324
São Miguel	SMG_0050_P1	Nordeste	Lomba da Fazenda	62	37.849901	-25.150251
	SMG_0050_P2			62	37.849915	-25.150426
	SMG_0200_P1	Povoação	Ribeira Quente	169	37.740682	-25.303525
	SMG_0200_P2			165	37.740648	-25.303084
	SMG_0400_P1		Lomba do Botão	462	37.774053	-25.275086
	SMG_0400_P2			460	37.773863	-25.275239
	SMG_0600_P1	Nordeste	Tronqueira	591	37.799094	-25.183596
	SMG_0600_P2			587	37.799082	-25.183441
	SMG_0800_P1	Povoação	Salto do Cavalo	765	37.787788	-25.277236
	SMG_0800_P2			743	37.787547	-25.277206
	SMG_1000_P1	Nordeste	Planalto dos Graminhais	1023	37.809714	-25.214029
	SMG_1000_P2			1018	37.809691	-25.214274
Terceira	TER_0040_P1	Angra do Heroísmo	Ponta do Queimado	41	38.766448	-27.375391
	TER_0040_P2			37	38.766525	-27.375152
	TER_0200_P1		Serreta	241	38.759882	-27.363701
	TER_0200_P2			243	38.760104	-27.363874
	TER_0400_P1		Pico do Carneiro	384	38.764920	-27.351604
	TER_0400_P2			378	38.765255	-27.351658
	TER_0600_P1		Pico da Lagoinha	679	38.752462	-27.331529
	TER_0600_P2			681	38.752538	-27.331343
	TER_0800_P1		Lagoa do Pinheiro trail	833	38.750801	-27.322126
	TER_0800_P2			833	38.750847	-27.322056
	TER_1000_P1		Serra de Santa Bárbara	994	38.730496	-27.322057
	TER_1000_P2			990	38.730633	-27.321635

**Table 2.** Species checklist, colonisation status and island records. Islands: SMG - São Miguel; TER - Terceira; PIC - Pico; FLO - Flores. Colonisation status (CS): END - Azorean endemic; NAT - native; INT NAT - introduced naturalised; INV - invasive. X - present, X* - new island record (not listed for that island in Silva et al. (2010)).

Phylum	Class	Order	Family	Scientific Name	CS	SMG	TER	PIC	FLO
Tracheophyta	Selaginellopsida	Selaginellales	Selaginellaceae	*Selaginella kraussiana* (Kunze) A.Braun	NAT	X	X	X	X
	Polypodiopsida	Cyatheales	Culcitaceae	*Culcita macrocarpa* C.Presl	NAT	X	X	X	X
			Cyatheaceae	*Dicksonia antarctica* Labill.	INV	X			
		Hymenophyllales	Hymenophyllaceae	*Hymenophyllum tunbrigense* (L.) Sm.	NAT	X	X	X	X
				*Hymenophyllum wilsonii* Hook.	NAT		X		X
				*Vandenboschia speciosa* (Willd.) G.Kunkel	NAT			X	
		Osmundales	Osmundaceae	*Osmunda regalis* L.	NAT	X		X	X
		Polypodiales	Aspleniaceae	*Asplenium hemionitis* L.	NAT				X
				*Asplenium marinum* L.	NAT			X	
				*Asplenium obovatum* Viv.	NAT			X	X
				*Asplenium onopteris* L.	NAT		X		
			Athyriaceae	*Deparia petersenii* (Kunze) M.Kato	INT NAT	X			
				*Diplazium caudatum* (Cav.) Jermy	NAT				X
			Blechnaceae	*Struthiopteris spicant* (L.) Weis	NAT	X	X	X	X
				*Woodwardia radicans* (L.) Sm.	NAT	X			X
			Dennstaedtiaceae	*Pteridium aquilinum* (L.) Kuhn	NAT	X	X	X	X
			Dryopteridaceae	*Cyrtomium falcatum* (L.f.) C.Presl	INV	X		X	X
				*Dryopteris aemula* (Aiton) Kuntze	NAT	X	X	X	X
				*Dryopteris affinis* (Lowe) Fraser-Jenk.	NAT	X			
				*Dryopteris azorica* (Christ) Alston	END	X	X	X	X
				*Dryopteris crispifolia* Rasbach, Reichst. & Vida	END	X			
				*Elaphoglossum hirtum* (Sw.) C.Chr.	NAT	X	X	X	X
				*Polystichum setiferum* (Forssk.) T.Moore ex Woynar	NAT	X			
			Polypodiaceae	Polypodium macaronesicum subsp. azoricum (Vasc.) Rumsey, Carine & Robba	END	X	X	X	X
			Pteridaceae	*Adiantum hispidulum* Sw.	INV	X			
				*Pteris incompleta* Cav.	NAT				X
			Thelypteridaceae	*Christella dentata* (Forssk.) Brownsey & Jermy	INT NAT	X		X	
				*Oreopteris limbosperma* (All.) Holub	NAT			X	
	Pinopsida	Pinales	Cupressaceae	*Cryptomeria japonica* D.Don	INT NAT	X	X		
				*Juniperus brevifolia* (Hochst. ex Seub.) Antoine	END	X	X	X	X
	Magnoliopsida	Apiales	Apiaceae	*Angelica lignescens* Reduron & Danton	END			X	
				*Sanicula azorica* Guthnick ex Seub.	END			X	
			Araliaceae	*Hedera azorica* Carrière	END	X	X	X	X
			Pittosporaceae	*Pittosporum undulatum* Vent.	INV	X	X	X	X
		Aquifoliales	Aquifoliaceae	*Ilex azorica* Gand.	END	X	X	X	X
		Asterales	Asteraceae	*Leontodon filii* (Hochst. ex Seub.) Paiva & Ormonde	END	X			
				*Leontodon longirostris* (Finch & P.D.Sell) Talavera	NAT			X	
				*Leontodon rigens* (Aiton) Paiva & Ormonde	END	X			
				*Pericallis malvifolia* (L’Hér.) B.Nord.	END	X			
				*Solidago azorica* Hochst. ex Seub.	END				X
				*Tolpis azorica* (Nutt.) P.Silva	END			X	
		Boraginales	Boraginaceae	*Myosotis maritima* Hochst. ex Seub.	END		X		
				*Sagina maritima* Don	NAT		X		
		Caryophyllales	Caryophyllaceae	Silene uniflora subsp. cratericola (Franco) Franco	END			X	
			Phytolaccaceae	*Phytolacca americana* L.	INV	X		X	X
		Commelinales	Commelinaceae	*Tradescantia fluminensis* Vell.	INV	X		X	
		Dipsacales	Adoxaceae	*Viburnum treleasei* Gand.	END	X	X	X	X
			Caprifoliaceae	*Leycesteria formosa* Wall.	INV	X			
		Ericales	Clethraceae	*Clethra arborea* Aiton	INV	X			
			Ericaceae	*Calluna vulgaris* (L.) Hull	NAT	X	X	X	X
				*Daboecia azorica* Tutin & E.F.Warb.	END			X	
				*Erica azorica* Hochst. ex Seub.	END	X	X	X	X
				*Vaccinium cylindraceum* Sm.	END	X	X	X	X
			Myrsinaceae	*Myrsine retusa* Aiton	END	X	X	X	X
			Primulaceae	*Lysimachia azorica* Hornem. ex Hook.	END	X	X	X	X
		Fabales	Fabaceae	*Acacia melanoxylon* R.Br.	INV	X			
				*Lotus parviflorus* Desf.	INT NAT	X			
		Fagales	Myricaceae	*Morella faya* (Aiton) Wilbur	NAT	X	X	X	X
		Gentianales	Gentianaceae	*Centaurium scilloides* (L.f.) Samp.	END	X	X		
			Rubiaceae	*Rubia agostinhoi* Dansereau & P.Silva	END	X		X	
		Lamiales	Lamiaceae	*Thymus caespititius* Brot.	NAT			X	
			Oleaceae	*Picconia azorica* (Tutin) Knobl.	END	X	X	X	X
			Plantaginaceae	*Plantago coronopus* L.	NAT	X			
				*Plantago lanceolata* L.	INT NAT		X		
			Scrophulariaceae	*Sibthorpia europaea* L.	NAT	X	X	X	X
		Laurales	Lauraceae	*Laurus azorica* (Seub.) Franco	END	X	X	X	X
				*Phoebe indica* Pax	INT NAT		X		
		Malpighiales	Euphorbiaceae	*Euphorbia azorica* Hochst. ex Seub.	END			X	
				*Euphorbia stygiana* H.C.Watson	END			X	
			Hypericaceae	*Hypericum foliosum* Aiton	END	X	X	X	X
			Violaceae	Viola palustris subsp. juressi (Link ex Cout.) Cout.	NAT			X	X
		Malvales	Thymelaeaceae	*Daphne laureola* L.	NAT			X	
		Myrtales	Myrtaceae	*Metrosideros excelsa* Gaertn.	INV		X*		
				*Psidium cattleyanum* Sabine	INT NAT		X*		X
		Ranunculales	Papaveraceae	*Fumaria muralis* W.D.J.Koch	INT NAT			X	
		Rosales	Moraceae	*Ficus carica* L.	INT NAT				X
			Rhamnaceae	*Frangula azorica* Grubov	END	X	X	X	X
			Rosaceae	*Fragaria vesca* L.	NAT			X	
				*Potentilla anglica* Laichard.	NAT		X		
				*Potentilla erecta* (L.) Raeusch.	NAT			X	
				*Potentilla* sp.	NAT	X			X
				Prunus lusitanica subsp. azorica (Mouill.) Franco	END	X			
				*Rubus hochstetterorum* Seub.	END			X	X
				*Rubus ulmifolius* Schott	INV	X	X	X	
		Santalales	Santalaceae	*Arceuthobium azoricum* Wiens & Hawksw.	END			X	
		Saxifragales	Crassulaceae	*Umbilicus horizontalis* (Guss.) DC.	NAT		X	X	
				*Umbilicus rupestris* (Salisb.) Dandy	NAT		X	X	
		Solanales	Solanaceae	*Physalis peruviana* L.	INT NAT			X	
				*Salpichroa origanifolia* (Lam.) Baill.	INV			X*	
				*Solanum mauritianum* Scop.	INV	X			
	Liliopsida	Alismatales	Araceae	*Zantedeschia aethiopica* Spreng.	INV			X	
		Asparagales	Iridaceae	*Iris foetidissima* L.	INT NAT	X			
			Orchidaceae	*Platanthera pollostantha* R.M.Bateman & M.Moura	END	X	X	X	
		Liliales	Smilacaceae	*Smilax azorica* H.Schaef. & P.Schönfelder	END	X		X	
		Poales	Cyperaceae	*Carex echinata* Murray	NAT		X		X
				*Carex hochstetteriana* J.Gay ex Seub.	END	X	X	X	X
				*Carex leviosa* Míguez, Jim.-Mejías, H. Schaef. & Martín-Bravo	END				X
				*Carex peregrina* Link	NAT	X		X	
				Carex pilulifera subsp. azorica (J.Gay) Franco & Rocha Afonso	END	X	X		
				*Carex vulcani* Hochst. ex Seub.	END	X		X	
				*Eleocharis multicaulis* (Sm.) Desv.	NAT		X		
			Juncaceae	*Juncus effusus* L.	NAT				X
				*Luzula purpureosplendens* Seub.	END	X	X	X	X
			Poaceae	*Agrostis congestiflora* Tutin & E.F.Warb.	END	X		X	
				*Anthoxanthum odoratum* L.	INT NAT			X	
				*Arundo donax* L.	INV	X			
				*Brachypodium sylvaticum* (Huds.) P.Beauv.	NAT	X			
				*Deschampsia foliosa* Hack.	END	X	X	X	X
				*Festuca francoi* Fern.Prieto, C.Aguiar, E.Días & M.I.Gut	END			X	X
				*Festuca petraea* Guthnick ex Seub.	END	X	X		X
				*Holcus lanatus* L.	INV	X			
				*Holcus rigidus* Hochst. ex Seub.	END	X	X	X	X
				*Hordeum murinum* L.	INT NAT		X*		
		Zingiberales	Zingiberaceae	*Hedychium gardnerianum* Sheppard ex Ker-Gawl.	INV	X	X	X	X
